# Sensitization of spinal cord nociceptive neurons with a conjugate of substance P and cholera toxin

**DOI:** 10.1186/1471-2202-8-30

**Published:** 2007-05-10

**Authors:** Robert M Caudle, Andrew J Mannes, Jason Keller, Federico M Perez, Shelby K Suckow, John K Neubert

**Affiliations:** 1 Department of Oral and Maxillofacial Surgery and Diagnostic Sciences, University of Florida College of Dentistry, Gainesville, FL 32610, USA; 2 Department of Orthodontics, University of Florida College of Dentistry, Gainesville, FL 32610, USA; 3 Department of Neuroscience, University of Florida College of Medicine, McKnight Brain Institute, Gainesville, FL 32610, USA; 4 Pain and Neurosensory Mechanisms Branch, National Institutes of Dental and Craniofacial Research, National Institutes of Health, Bethesda, MD 20892, USA

## Abstract

**Background:**

Several investigators have coupled toxins to neuropeptides for the purpose of lesioning specific neurons in the central nervous system. By producing deficits in function these toxin conjugates have yielded valuable information about the role of these cells. In an effort to specifically stimulate cells rather than kill them we have conjugated the neuropeptide substance P to the catalytic subunit of cholera toxin (SP-CTA). This conjugate should be taken up selectively by neurokinin receptor expressing neurons resulting in enhanced adenylate cyclase activity and neuronal firing.

**Results:**

The conjugate SP-CTA stimulates adenylate cyclase in cultured cells that are transfected with either the NK1 or NK2 receptor, but not the NK3 receptor. We further demonstrate that intrathecal injection of SP-CTA in rats induces the phosphorylation of the transcription factor cyclic AMP response element binding protein (CREB) and also enhances the expression of the immediate early gene c-Fos. Behaviorally, low doses of SP-CTA (1 μg) injected intrathecally produce thermal hyperalgesia. At higher doses (10 μg) peripheral sensitivity is suppressed suggesting that descending inhibitory pathways may be activated by the SP-CTA induced sensitization of spinal cord neurons.

**Conclusion:**

The finding that stimulation of adenylate cyclase in neurokinin receptor expressing neurons in the spinal cord produces thermal hyperalgesia is consistent with the known actions of these neurons. These data demonstrate that cholera toxin can be targeted to specific cell types by coupling the catalytic subunit to a peptide agonist for a g-protein coupled receptor. Furthermore, these results demonstrate that SP-CTA can be used as a tool to study sensitization of central neurons in vivo in the absence of an injury.

## Background

Several groups have developed potential therapeutics that produce highly selective lesions in vivo by exploiting specific g-protein coupled receptors (GPCRs) as transporters to deliver a toxin to an intracellular target [1-9]. When GPCRs bind a peptide agonist they are internalized by the cell; delivering the peptide, and any attached toxin, to the inside of the cell [10]. The toxin is then able to act on its intracellular target. Some of these investigators have used lethal toxins such as saporin, diphtheria and pseudomonas exotoxin coupled to the neuropeptide substance P to target the agents to cells expressing neurokinin receptors [1,3,7-9,11]. These toxins produce highly specific lesions of neurokinin receptor expressing cells while not harming cells in the region that do not express these receptors. The investigators have also demonstrated by ablating these cells that neurons expressing the NK1 receptor in the spinal cord are required for central sensitization. Thus, these targeted toxins were found to be valuable tools for evaluating the function of neurons in the central nervous system [2-4,12]. Moreover, it has been suggested that these targeted toxins may have clinical utility for the treatment of intractable pain.

In an effort to compliment the armamentarium of targeted toxins we sought to selectively activate, rather than kill, neurokinin receptor expressing cells by coupling cholera toxin to the neuropeptide substance P. Cholera toxin, unlike previously used toxins, is not universally lethal to the cells. The toxin is useful because it ADP ribosylates the g-protein Gs, which results in the uncoupling of the protein from GPCRs and activation of the g-protein [13-16]. Cholera toxin activation of Gs stimulates adenylate cyclase activity to produce higher levels of cAMP in the cells, altered protein kinase activity and altered ion channel activity [13,16-21]. Thus, we hypothesized that a conjugate of substance P and the catalytic subunit of cholera toxin (SP-CTA) would selectively activate neurokinin receptor expressing neurons and would provide a novel tool for evaluating cell function in vivo.

## Results

### Synthesis of SP-CTA

**Figure 1:**
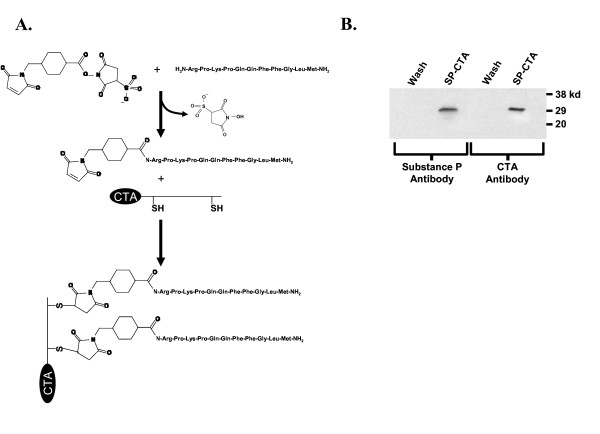
Synthesis of SP-CTA. **A**. Schematic representation of the procedure used to synthesize SP-CTA. **B**. Western blots of final SP-CTA product. SP-CTA and the filtrate from the Centricon Plus-20 concentrating tubes (Wash) were run on western blots and probed with antibodies to substance P and the catalytic subunit of cholera toxin (CTA). The SP-CTA product reacted with both antibodies.

The neuropeptide substance P was coupled to the catalytic subunit of cholera toxin (CTA) using the bifunctional linking agent sulfosuccinimidyl 4-N-maleimidomethyl cyclohexane-1-carboxylate (Sulfo-SMCC) as indicated in figure 1A. Briefly, the Sulfo-SMCC was reacted with the N-terminal amine of substance P to form an amide linkage to the maleimide group. The substance P – maleimide was then conjugated to CTA through two cysteine residues in the C-terminal region of the CTA protein. The final product was washed and concentrated by centrifugation in Centricon filters with a cutoff of 5 kd. The success of the synthesis was confirmed on western blots by using antibodies to both substance P and CTA. As demonstrated in figure 1B the final product produced bands on the western blot with a molecular weight of approximately 30 kd that reacted with antibodies to substance P and CTA indicating a successful coupling of substance P to CTA (SP-CTA). Based on protein assays the synthetic yields were quantitative. In preliminary syntheses bands for substance P and CTA in the western blots were doublets. Increasing the concentration of substance P in the reaction produced a single band at the higher molecular weight suggesting that the stoichiometry of substance P to CTA in the final product was 2:1.

### In situ evaluation of SP-CTA

**Figure 2:**
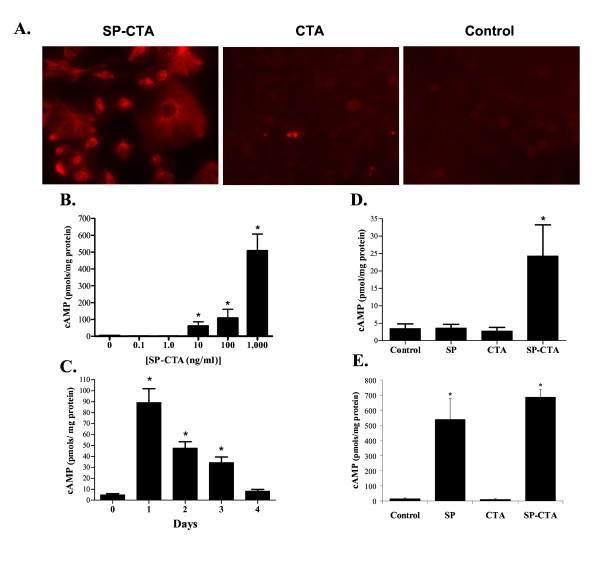
In situ evaluation of SP-CTA. **A**. CHO cells stably transfected with the NK1 receptor were treated with either SP-CTA (1 μg/ml), CTA (1 μg/ml) or were not exposed to any agents (Control). The cells were cultured over night in these solutions and then immunocytochemistry was performed on the cells using an antibody to CTA and Rhodamine-tyramide amplification. Labeling was observed only in the SP-CTA treated cells. **B**. Concentration response relationship for SP-CTA on cAMP production. CHO-NK1 cells were exposed to the indicated concentrations of SP-CTA for 1 hour. The cells were cultured for another 24 hours following washout of the SP-CTA with fresh culture media. The cells were harvested and cAMP content was measured using a commercial cAMP assay. (N = 5, 100 mm plates/concentration) (ANOVA F = 56.15, df = 83, p < 0.0001). **C**. Time course of SP-CTA's effects on cAMP content of CHO-NK1 cells. The cells were incubated for 1 hour with 100 ng/ml SP-CTA and cultured in fresh media for the indicated times. The cells were then harvested and analyzed for cAMP content. (N = 5, 100 mm plates/treatment) (ANOVA F = 25.35, df = 29, p < 0.0001). **D**. Comparison of the effects of substance P (SP), the catalytic subunit of cholera toxin (CTA) and SP-CTA on cAMP production in CHO-NK1 cells. Cells were incubated with 100 ng/ml of CTA, SP, SP-CTA or received no treatment for 4 hours. The cells were then cultured for 48 hours in fresh media, harvested and assayed for cAMP content. (N = 5, 100 mm plates/treatment) (ANOVA F = 5.27, df = 19, p = 0.0102) **E**. Further comparison of the effects of SP, CTA and SP-CTA on cAMP production. Cells were treated as described in D except the agents remained in the culture media for the full 48 hours. The cells were then harvested and analyzed for cAMP content. (N = 5, 100 mm plates/treatment) (ANOVA F = 23.06, df = 19, p < 0.0001). Data are expressed as means ± SEM. Asterisks indicate p < 0.05 one way ANOVA followed by Dunnett's test, F = 23.06, df = 19).

SP-CTA was tested on Chinese Hamster Ovary cells that were stably transfected with the NK1 receptor [22] (CHO-NK1). To verify selective uptake of SP-CTA by the cells, the cells were incubated over night in either SP-CTA (1 μg/ml) or CTA (1 μg/ml). The CHO-NK1 cells were then fixed and prepared for immunocytochemistry with antibodies to CTA using a rhodamine-tyramide amplification system. As illustrated in figure 2A only the SP-CTA treated cells demonstrated an uptake of CTA indicating that linkage of CTA to substance P was required for the conjugate to be internalized.

We further evaluated the functionality of the SP-CTA by examining the ability of the conjugate to stimulate cAMP production in CHO-NK1 cells. Figure 2B demonstrates the concentration response relationship for SP-CTA (0.1 ng/ml (3.4 pM) to 1,000 ng/ml (34 nM)) when the SP-CTA was applied for 1 hour and the cAMP was measured after culturing the cells for an additional 24 hours. The time course of SP-CTA's effect on cAMP production was evaluated by treating the cells for 1 hour with 100 ng/ml SP-CTA and then harvesting the cells for cAMP analysis 1, 2, 3 and 4 days following exposure to the conjugate. As demonstrated in figure 2C, SP-CTA's effects on cAMP peaked at 1 day in the CHO-NK1 cells and remained significantly elevated for 3 days.

Figure 2D demonstrates that 48 hours following a 4 hour exposure to 100 ng/ml of either substance P, CTA or SP-CTA only SP-CTA treated CHO-NK1 cells produced an increase in cAMP production. When 100 ng/ml of substance P, CTA or SP-CTA remained in the culture media for the full 48 hours the substance P treated cells and the SP-CTA treated cells had significantly elevated levels of cAMP, whereas the CTA treated cells did not differ from control cells (Figure 2E). The findings illustrated in figures 2D and 2E indicate that substance P activation of NK1 receptors can stimulate adenylate cyclase, but that the effects of SP-CTA on cAMP production outlast any stimulation of the NK1 receptors produce by the substance P portion of the conjugate when the material is washed out of the culture media. The ability of substance P to stimulate cAMP production was previously demonstrated in several different cell types including CHO-NK1 cells [23-25], which is consistent with our findings.

**Figure 3:**
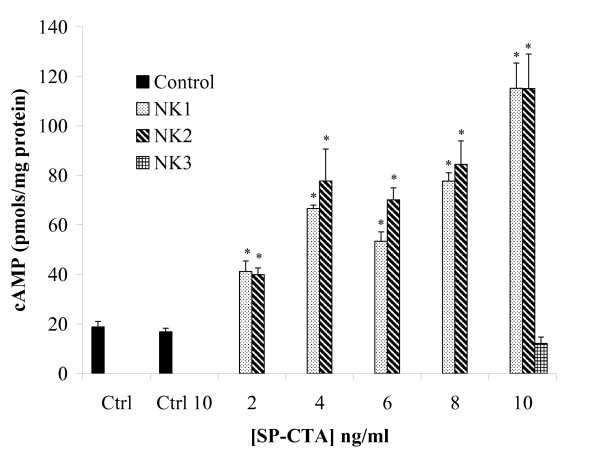
Relative selectivity of SP-CTA for NK1, NK2 and NK3 receptors. Concentration response relationships for SP-CTA on the stimulation of cAMP production were performed on CHO cells transiently transfected with NK1 or NK2 receptors. Non-transfected CHO cells were either not exposed to SP-CTA (Ctrl) or treated with 10 ng/ml SP-CTA (Ctrl 10). CHO cells stably transfected with NK3 receptors were treated with a single concentration of 10 ng/ml SP-CTA. (Two way ANOVA of NK1 versus NK2, F = 1.7, df = 1, p = 0.20: Dose, F = 54.61, df = 6, p < 0.0001). Asterisks indicate p < 0.01 (one way ANOVA followed by Dunnett's post hoc test, F = 56.15, df = 83) when compared to control cells not treated with SP-CTA.

To evaluate the relative selectivity of SP-CTA for NK1, NK2 and NK3 receptors, CHO cells were transfected with NK1 or NK2 plasmids and a concentration response relationship was performed on SP-CTA's ability to stimulate cAMP production. As demonstrated by figure 3, cells containing NK1 or NK2 receptors demonstrated equivalent concentration response relationships when measuring levels of cAMP stimulation by SP-CTA. However, in CHO cells not expressing neurokinin receptors or CHO cells stably expressing NK3 receptors SP-CTA did not stimulate cAMP production (Figure 3).

### In vivo evaluation of SP-CTA

**Figure 4:**
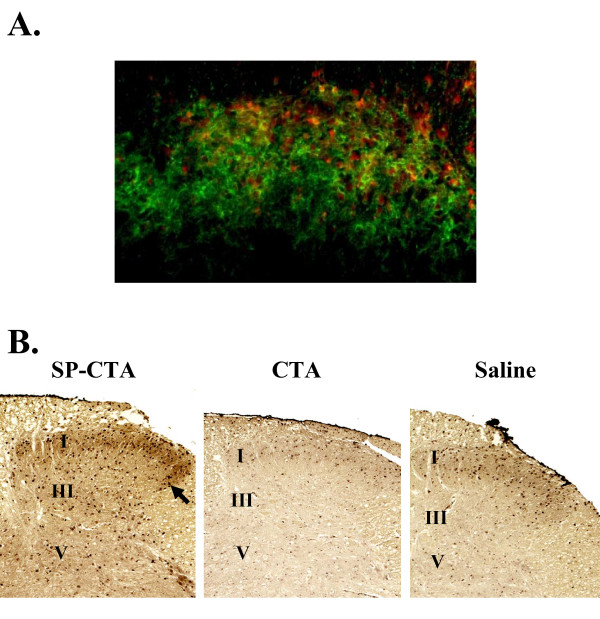
Intrathecal administration of SP-CTA **A**. NK1 expressing neurons in the dorsal horn take up SP-CTA. Three rats received intrathecal injections of 10 μg SP-CTA. One hour following the injections the animals were euthanized and the spinal cords were removed and labeled for NK1 (green) and CTA (red) using immunohistochemistry. Cells co-labeled for NK1 and CTA appear yellow to orange. These data indicated that SP-CTA was taken up by the NK1 receptor expressing neurons.** B**. Activation of dorsal horn neurons by SP-CTA. Rats (N = 6 per treatment group) received intrathecal injections of SP-CTA (50 μg), CTA (50 μg) or Saline (20 μl). The animals were allowed to recover for 24 hours. The animals were euthanized and perfused as described in the methods section. The spinal cords were removed and prepared for immunohistochemistry using an antibody to pCREB, a secondary antibody coupled to HRP and diaminobenzidine. Phosphorylated CREB is visible as dark staining nuclei. The arrow points to an example of pCREB expressing nuclei. The approximate locations of lamina I, III and V are indicated. These data indicate that CREB phosphorylation is stimulated by SP-CTA, but not CTA alone.

**Figure 5:**
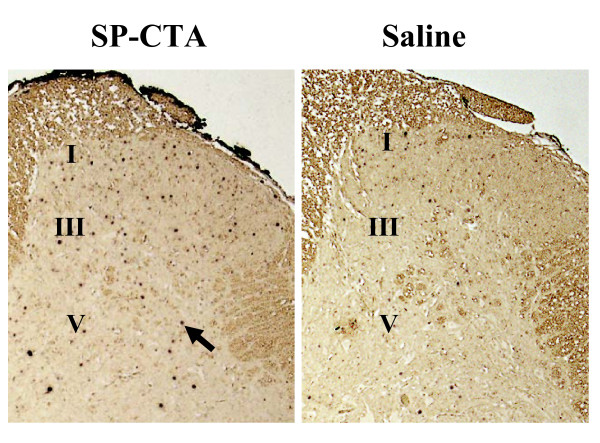
SP-CTA stimulates the production of the immediate early gene c-Fos in dorsal horn neurons. Sections from the same animals as in figure 4 were used for immunohistochemistry with antibodies to c-Fos. The sections were prepared as previously described. The c-Fos labels as dark staining nuclei. The arrow points to an example of a c-Fos positive nucleus. The approximate locations of lamina I, III and V are indicated. These data indicate that c-Fos expression is stimulated by SP-CTA.

Previous work with lethal toxins coupled to substance P demonstrated that neurons expressing NK1 receptors are necessary for the expression of thermal hyperalgesia [1,3,7-9]. Therefore it was hypothesized that uptake of SP-CTA by NK1 receptor expressing neurons in the spinal cord would stimulate the cells and produce thermal hyperalgesia. Initially, a group of 3 rats received intrathecal injections of 10 μg of SP-CTA. One hour later the spinal cords were harvested and prepared for immunohistochemistry to determine if the SP-CTA was taken up by NK1 receptor expressing neurons. Figure 4A demonstrates that the conjugate co-labels with NK1 receptors in the superficial layers of the spinal cord. To test the idea that SP-CTA would stimulate the neurons that have taken up the conjugate rats were injected intrathecally with SP-CTA (50 μg), CTA (50 μg) or saline (20 μl) (N = 6 per treatment group). Twenty four hours following the injection of SP-CTA, but not CTA, the animals were agitated and aggressive toward their cage mates. The animals were euthanized by pentobarbital overdose and prepared for immunohistochemistry of the spinal cords to evaluate the phosphorylation of cAMP response element-binding protein (CREB) and the expression of the immediate early gene c-Fos. We chose to examine CREB because elevated levels of cAMP lead to the phosphorylation of this transcription factor [26]. We found that SP-CTA treatment resulted in a large increase in the phosphorylation of CREB in the spinal cord dorsal horn (Figure 4B), while the injection of CTA produced levels of phosphorylated CREB that were similar to saline injected animals. In addition to elevated levels of phosphorylated CREB we found that c-Fos was also enhanced in SP-CTA treated animals (Figure 5).

**Figure 6:**
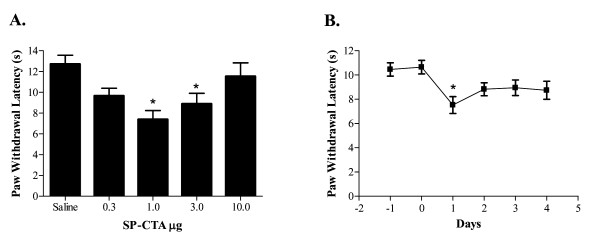
Effect of intrathecal SP-CTA on thermal nociception in rats. **A**. SP-CTA dose response relationship. Rats (N = 10/dose) received intrathecal injections of the indicated doses of SP-CTA in 20 μl of saline. Twenty four hours following the injections the animals were tested for thermal nociception using a Hargreaves apparatus [27]. Asterisks indicate p < 0.05 one way ANOVA (F = 5.527, df = 49) followed by Dunnett's test when compared to the saline treated animals. **B**. Time course of intrathecal SP-CTA on thermal nociception. Rats (N = 20) were injected intrathecally with 1 μg of SP-CTA in 20 μl of saline. The animals were tested for thermal nociception at the times indicated. Zero represents the day of injection. Data are presented as means ± SEM. Asterisk indicates p < 0.05 repeated measures ANOVA (F = 5.06, df = 99) followed by Dunnett's test when compared to the zero time point.

A dose response relationship and time course was determined for SP-CTA using the hind paw thermal nociception assay of Hargreaves et al. [27]. Figure 6A demonstrates that intrathecally administered SP-CTA (N = 10 rats per dose) has a biphasic dose response relationship 24 hours following the injections with peak thermal hyperalgesia observed at a dose of approximately 1 μg (34 pmoles). At 10 μg of SP-CTA the animals demonstrated agitated behaviors similar to the first group that received 50 μg; therefore, no higher doses were tested. Finally, to determine the time course of action of SP-CTA 1 μg was injected intrathecally (N = 20 rats) and thermal nociception was tested before injections and 1 to 4 days following the injection. As illustrated in figure 6B the peak of thermal hyperalgesia occurred 1 day following the injection. Some recovery occurred over the next three days; however, a complete return to baseline paw withdrawal thresholds was not observed.

## Discussion

Previously used lethal toxins targeted to GPCRs via coupling to a peptide agonist were useful for evaluating the function of the cells that expressed the GPCRs. By using the receptor to direct the toxins to the desired cell type the agents produced highly specific lesions, even when the targeted cells were a minor constituent of a heterogeneous population of cells [1-9,11,12]. However, because these toxins kill the targeted cells, the cells' function must be inferred by the deficit that is produced by the lesion. Ideally, a method to selectively stimulate the cells could provide more information about the cells' function. To achieve this goal we coupled the neuropeptide substance P, which targets neurokinin receptors [2,3,8], to the catalytic subunit of cholera toxin (SP-CTA). Cholera toxin is not lethal when taken up by cells, but it does enhance the activity of adenylate cyclase resulting in an increase in intracellular cAMP [16]. Thus, by targeting cholera toxin to neurokinin receptor bearing neurons with substance P we altered the function of these cells rather than killing them.

We found that SP-CTA produced equivalent effects on cAMP production in cultured cells expressing NK1 or NK2 receptors, but did not influence cAMP production in cells that expressed NK3 receptors or non-transfected cells. These data suggest that the conjugate binds to NK1 and NK2 receptors with approximately the same affinity. Takeda and colleagues previously demonstrated that substance P had a ten fold higher affinity for NK1 than NK2 receptors [25]. It is possible that the presence of CTA on the N terminus of substance P reduces the NK1 selectivity of the peptide. Alternatively, since we used a functional assay, any affinity differences between the receptors may have been obscured by uptake mechanisms and by the action of the toxin on Gs. For example, the association rate of substance P is much higher for NK1 than NK2 receptors [25]. Because of the higher binding rate the NK1 receptors may be internalized faster than the NK2 receptors. However, if the NK1 receptors do not rapidly recycle to the cell surface the NK2 receptors will eventually bind and internalize an equal amount of SP-CTA. Thus our functional assay would negate the differences in binding affinity of substance P for the NK1 and NK2 receptors. Additionally, because cholera toxin stimulates cAMP production and protein kinase A activity it is possible that due to excessive phosphorylation the neurokinin receptors are trapped in the internalized state making the receptors unavailable to transport more toxin. This process could also mask any binding affinity differences.

In vivo our data demonstrate that when SP-CTA was injected intrathecally in rats the animals became hypersensitive to thermal stimuli (Figure 6A and 6B). This data supports the previous lesion studies indicating that central sensitization is mediated by NK1 receptor expressing neurons in the spinal cord [1-3,6]. We further found that with higher doses of SP-CTA peripheral sensitivity was suppressed (Figure 6A). However, the animals demonstrated behaviors that suggested centrally mediate nociception. We hypothesize that the suppression of peripheral hypersensitivity was due to stimulation of inhibitory pathways by the NK1 receptor expressing neurons that have taken up the SP-CTA. These inhibitory systems may be previously characterized descending inhibitory pathways [28] or local inhibitory neurons. Further work should clarify this finding. These data, however, support the idea that selectively stimulating cells provides unique information on their function that is not available when a lesioning strategy is used. What is most notable about these results is that the inhibition of peripheral nociception could not have been predicted from several previous lesioning studies [1-3,6]. However, when specifically examining descending inhibition Suzuki and colleagues' found that lesioning of NK1 expressing neurons in the spinal cord suppressed peripheral afferent stimulated descending inhibition [29], which is consistent with our findings.

The effects of intrathecally administered SP-CTA also contrast with the effects produced by intrathecal wild type cholera toxin. In a previous study wild type cholera toxin inhibited the hyperalgesia and allodynia produced by a variety of peripheral injuries [30]. The inhibitory effect of the toxin was blocked by the opioid antagonist naloxone. The mechanism that produced this endogenous opioid mediated inhibition of nociception was not investigated, but work by Shen and Crain indicates that cholera toxin can enhance the inhibitory effects of opioids on primary afferent neurons [18]. Thus, it can be hypothesized that wild type cholera toxin acts principally on primary afferent neurons to inhibit peripherally mediated nociception; while SP-CTA affects NK1 receptor expressing spinothalamic tract neurons to generate centrally mediated nociception.

## Conclusion

We have synthesized a novel tool for activating adenylate cyclase in neurokinin receptor expressing cells to evaluate the function of these cells in heterogeneous populations, as are typically found in vivo. Intrathecal injections of SP-CTA in rats demonstrated that the toxin conjugate produced thermal hyperalgesia as would be expected if NK1 receptor expressing spinothalamic tract neurons were sensitized by the treatment. These data indicate that GPCRs can be exploited to transport cholera toxin into a host of different cell types. Because the toxin is targeted to the GPCR by a peptide agonist it is remarkably simple to change the peptide to direct the conjugate to receptors other than neurokinin receptors. These targeted cholera toxin conjugates could find utility in a number of biomedical research endeavors.

## Methods

### Synthesis of SP-CTA

The A subunit of cholera toxin (CTA) was purchased from List Biological Laboratories inc. (Cambell, CA, USA). CTA has two cysteine residues in the C-terminal region [15,31] therefore these cysteine residues were used to attach substance P to CTA. The synthesis of SP-CTA was accomplished using a modification of Pierce Biotechnology inc.'s maleimide protein cross-linking procedure. The synthesis was carried out in two stages. The first stage was to link maleimide to the N-terminus of substance P by combining a 5 fold excess of Sulfosuccinimidyl 4-N-maleimidomethyl cyclohexane-1-carboxylate (Sulfo-SMCC) with substance P in phosphate buffered saline (PBS, pH 7.4). The mixture was incubated at room temperature for 1 hour. The substance P maleimide conjugate was separated from unreacted Sulfo-SMCC using a Sephadex G-10 (30 × 1.5 cm) column eluted with PBS. For the second phase of the synthesis the substance P maleimide conjugate was linked to the two cysteines on CTA by adding a 10 fold excess of the conjugate to CTA in PBS. This mixture was then incubated at room temperature for another hour. The SP-CTA was separated from the unreacted substance P maleimide conjugate, washed with PBS three times and concentrated using Centricon Plus-20 filters. A sample of the final product was evaluated by western blots. Briefly, the sample was run on 4–20% polyacrylamide electrophoresis gels, transferred to Polyvinylidene fluoride (PVDF) membranes and then probed with antibodies to either substance P or the catalytic subunit of cholera toxin. A secondary antibody coupled to horse radish peroxidase (HRP) and enhanced chemiluminescence were used to visualize the bands. Figure 1 illustrates the synthetic pathway as well as western blots of the final product.

### Cell culture

Chinese Hamster Ovary cells stably expressing NK1, NK2 or NK3 receptors (a generous gift from Dr. James Krause, Neurogen Corp. [22]) were plated on 100 mm plates for cAMP assays or 13 mm cover slips in 24 well culture plates for immunocytochemistry experiments. The cultures were grown in F12K media, 10% Fetal Bovine Serum, 1% L-glutamine, 1% penicillin-Streptomyosin, 25 mM Hepes buffer, and G418 (500 μg/ml). The cells were cultured at 37°C in a 5% CO2 atmosphere. Additionally, plasmids containing either NK1 or NK2 receptors were purchased (UMR cDNA Resource Center, Rolla, MO, USA) and transfected into CHO cells using Lipofectamine (Invitrogen, Carlsbad, CA, USA) as per the manufacturer's instructions. These cells were cultured as described for the stably transfected cells.

### cAMP assay

To assay cAMP levels in the cell cultures Sigma inc's (St. Louis, MO, USA) Direct cAMP Enzyme Immunoassay was used according to the manufacturer's instructions. Briefly, the media on the cell cultures was removed and the cells were washed once with PBS (pH 7.4). The PBS was removed and 1 ml 0.1 M HCL was added to the cells. The cells were scraped from the plates into the HCL solution, sonicated and centrifuged (600 g, 10 minutes, 5°C) and the supernatant collected. The protein in each sample was measured using Bio-Rad's (Hercules, CA, USA) protein assay. The cAMP was acetylated with the addition of 100 μl of the kit's acetic anhydride solution. A 100 μl sample was then neutralized with 50 μl of the kit's neutralizing buffer and the samples were added to the kit's 96 well plates that were pre-absorbed with antibodies to cAMP. A standard curve and controls were set up as suggested by the manufacturer. A cAMP- alkaline phosphatase conjugate (50 μl) was added to the wells and the solution was incubated for 2 hours. The plates were then washed 3 times and 200 μl of p-nitrophenyl phosphate solution (substrate) was added to each well and the plates were incubated for 1 hour. The reaction was stopped with 50 μl 0.1 M HCL and the plate was read at 405 nm. The concentration of cAMP in the samples was extrapolated from the data collected for the cAMP standards and expressed as the number of moles of cAMP per mg protein.

### Animals

Male Sprague Dawley rats (200 – 300 g) were housed in pairs and supplied standard rat chow and water *ad libitum *in the University of Florida's vivarium, which is an AAALAC certified facility.

Intrathecal injections were performed under isoflurane anesthesia via lumbar puncture between L4 and L5. All animal procedures in this project were reviewed and approved by the University of Florida's Institutional Animal Care and Use Committee.

### Immunocytochemistry and immunohistochemistry

Rats were euthanized with pentobarbital and immediately transcardially perfused with ice cold PBS and then ice cold 4% paraformaldehyde in phosphate buffered saline (PBS)(pH 7.4). Cell cultures on cover slips were washed with PBS and fixed with 4% paraformaldehyde in PBS. The spinal cords were removed and post fixed overnight in 4% paraformaldehyde in PBS. The tissue was cryoprotected in 30% sucrose, mounted and sectioned in a cryostat (-20°C)(10–20 μm) and mounted on slides. The sections or cells were then blocked with 3% normal goat serum for 60 minutes with 0.75% triton X-100. The primary antibody was added to the blocking solution (1:500) and the sections were incubated for 48 hours at 4°C. The sections or cells were then washed (8 × 5 mins) in PBS. Following the wash the sections or cells were incubated for 1 hour at room temperature in PBS with a secondary antibody that was coupled to horse radish peroxidase (HRP), Alexa Fluro 594 or Alexa Fluro 488 (Molecular Probes, Boston, MA). The sections labeled with the Alexa Fluro stains were washed (8 × 5 mins) and viewed using fluorescence microscopy. The HRP label sections were washed similarly and treated with diaminobenzidine. The cultured cells, which were treated with HRP coupled secondary antibodies, were washed similarly and labeled using rhodamine labeled tyramide as described by the manufacturer (NEN, Boston, MA, USA). Antibodies to c-Fos were obtained from Dr. Michael Iadarola (National Institute of Dental and Craniofacial Research, National Institutes of Health) [32], antibodies to CTA were purchased from Sigma (St. Louis, MO), antibodies to pCREB were purchased from New England Biolabs (Ispwich, MA) and antibodies to NK1 receptor were purchased from Zymed (San Francisco, CA).

### Thermal nociception assay

Thermal nociception was measured using the method of Hargreaves et al. [27]. Briefly, the rats were placed on a clear glass surface and allowed 15 minutes to accommodate to the enclosure. An infrared light was directed onto a hind paw's plantar surface approximately in the middle of the foot. The latency for the animal to remove its foot from the path of the light was used as the dependent measure for thermal sensitivity. The test was performed 4 times for each time point (2 tests for each hind foot) with each test separated by 2 minutes to prevent paw sensitization. The withdrawal latencies of the tests were averaged to obtain the final value for that time point.

### Statistics

Data were analyzed using a one way ANOVA followed by Dunnett's post-hoc test, one way repeated measures ANOVA or two way ANOVA as appropriate. Significance was assigned to p ≤ 0.05.

## Authors' contributions

*RMC *was the principle investigator on the project, synthesized the SP-CTA and performed behavioral experiments. *AJM, SKS *and *JK *performed the immunohistochemistry on the spinal cord tissue. *FMP *performed the cell culture and cAMP experiments. *JKN *assisted in experimental design and statistical analysis.
